# Is sleep disturbance in patients with chronic pain affected by physical exercise or ACT-based stress management? – A randomized controlled study

**DOI:** 10.1186/s12891-018-2020-z

**Published:** 2018-04-10

**Authors:** Tobias Wiklund, Steven J. Linton, Peter Alföldi, Björn Gerdle

**Affiliations:** 1 Pain and Rehabilitation Centre, and Department of Medical and Health Sciences, Linköping University, Linköping, Sweden; 2 CHAMP, School of Law, Psychology and Social Work, Örebro University, Örebro, Sweden; 3 Department of Medical and Health Sciences, University of Linköping, SE-581 85 Linköping, Sweden

**Keywords:** Sleep, Insomnia, Rehabilitation, Chronic pain, Exercise, Acceptance and commitment therapy, Randomized controlled trial

## Abstract

**Background:**

Most people suffering chronic pain are plagued by sleeping difficulties. Cognitive behaviour therapy has produced promising results for insomnia comorbid with chronic pain, but the access to such treatment is often limited. Over the last ten years, interventions aiming to increase cognitive flexibility and physical activity have been assumed to be effective treatments for a variety of conditions, including insomnia and chronic pain. If proven effective, these treatments could constitute the first steps in a stepped care model for chronic pain and insomnia.

**Methods:**

Two hundred ninety-nine chronic pain subjects were randomized to Exercise, ACT-based stress management (ACT-bsm), or an active control group. Two hundred thirty-two participants (78%) received their allocated intervention at least to some extent. These participants were evaluated using mixed model analyses for changes in sleep (Insomnia Severity Index, ISI), pain intensity, depression, and anxiety immediately after treatment, six months and twelve months after treatment.

**Results:**

The mixed model analyses revealed that Exercise had a positive effect on insomnia compared with the control group and the effect remained after 12 months. No clear effect (i.e., both for completers and for completers together with treatment non-completers) upon ISI was found for the ACT-bsm. Pain intensity decreased significantly both in the exercise group and in the control group. For the two psychological variables (i.e., symptoms of anxiety and depression) were found significant improvements over time but no group differences. The treatment effects for ISI and pain intensity did not reach clinical significance per definitions presented in other relevant studies.

**Conclusions:**

Beneficial significant effects on insomnia was confirmed in the exercise condition. However, these changes were probably not clinically important. For pain intensity a general decrease was found in the Exercise condition and in the control condition, while no change occurred in ACT-bsm. No group differences were found for the two psychological variables.

**Trial registration:**

The study was registered in Clinical Trials (Trial registration: ClinicalTrials.gov Id: NCT02399644, 21 January 2015, retrospectively registered).

## Background

Most people suffering chronic pain are also plagued by sleeping difficulties. Chronic moderate/severe pain among adults has a prevalence of nearly 20% [1]. Moderate to severe insomnia (score ≥ 15) according to the Insomnia Severity Index (ISI) is frequently (53–65%) reported among patients with chronic pain [2–4]. Although chronic pain conditions are the third most common reason for sick leave in Sweden, there are few effective treatments for chronic pain [5]. Hypnotics are commonly used as treatment for sleep disturbances, but for many reasons they are recommended only for short-term use [6].

Physical exercise has been investigated as treatment for sleeping problems in populations such as older adults and non-clinical samples. Kline [7], in a review of the literature, concluded that the three studies that exclusively included patients diagnosed with chronic insomnia had the most robust results regarding insomnia [8–10]. Exercise is a common component of multimodal/multidisciplinary rehabilitation programmes (MMRP) for chronic pain patients, but the effects of this component have rarely been evaluated with respect to sleep. One exception is Eadie, et al. [11] who randomized chronic low back pain patients with sleep disturbance (*N* = 60) into three different forms of physiotherapy/exercise including an active control group. They found medium size effects in all groups according to ISI and Pittsburgh Sleep Quality Index.

Growing evidence suggests that an effective treatment for sleeping problems comorbid with chronic pain could be Cognitive Behavioural Therapy for Insomnia (CBT-I) [12–14]. However, CBT-I requires trained clinicians and is rather work intensive, so access to CBT-I is very limited. Stress management training is another candidate since it addresses psychological flexibility; i.e., the ability to make conscious decisions when aware of your inner state and the outer world. By fostering a non-judgmental approach to these experiences, the patient can lower the impact of rule-governed behaviours and thus act in a more goal-oriented manner [15]. Cognitive flexibility had a mediating role in effective pain rehabilitation [16, 17]. It has also been proposed as an important process in primary insomnia [18, 19] and even in insomnia comorbid with chronic pain [20]. Flaxman, et al. [21] developed a workplace intervention to enhance psychological flexibility and wellbeing based on the principles of Acceptance and Commitment Therapy (ACT). Livheim developed a Swedish version of Flaxman’s protocol, which here will be labelled as ACT-based stress management (ACT-bsm). ACT-bsm is a generic course administrated by any health professional with training in the method thus increasing availability in e.g., primary care. ACT-bsm does not include elements such as behavioural analyses or individualised interventions. ACT-bsm, although implemented without supporting evidence, has increasingly been used in Sweden for patients with pain conditions.

In the search for accessible and effective treatments, there is a need to evaluate physical exercise and ACT-bsm as unimodal interventions with respect to their effects on insomnia, pain and depressive and anxiety symptoms in patients with chronic pain conditions. We hypothesised that physical exercise and ACT-bsm had positive effects upon insomnia, pain and psychological symptoms. To this end, we compared group-based ACT-bsm, group-based *physical exercise* (Exercise), and group-based *discussion* concerning pain and its consequences (i.e., an active control group, CON).

## Methods

### Procedure and study design

**Fig. 1:**
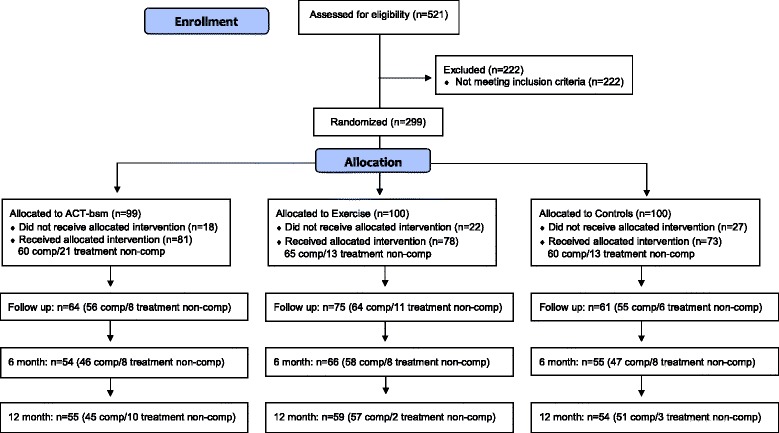
Participant flowchart. Comp = completer

This study is part of the randomized controlled trial FACTA, which was performed at Linköping University Hospital (Linköping, Sweden). The main study – according to the registration in Clinical Trials (see below) - will be presented elsewhere. Adults with chronic benign neck, low back, and/or generalized pain were included. The participants (*n* = 299) were randomized to Exercise, ACT-bsm, or CON. Treatments lasted between seven and eight weeks and all participants were measured before and after treatment and followed up at six and twelve months (Fig. 1).

### Subjects

Adults (18–60 years) with chronic (> 3 months) benign neck, low back, and/or generalized pain were included. Severe psychiatric, neurological, endocrinological, cardiovascular, and inflammatory diseases were excluded (i.e., diseases that required other treatments or that would hinder participation in the Exercise intervention). Poor skills in Swedish language (i.e., difficulties in understanding spoken and/or written Swedish, which would hinder full participation in ACT-bsm or CON) and a very stressful social situation (e.g., ongoing abuse or lack of permanent housing) were also general exclusion criteria.

Participants were recruited from three different sources. Originally, records from the Pain and Rehabilitation Centre, University Hospital, Linköping were screened for patients with chronic back and/or neck pain. This screening resulted in 496 patients being sent a request to participate. Of these 496, 110 patients responded and were assessed by a telephone interview. Those who met the inclusion criteria were assessed in the clinic for the medical inclusion criteria. This assessment yielded 67 candidates, and these were randomized into the three groups. Since the design of the study required far more participants, an additional application was sent to the ethical board asking for approval to use print advertisement to recruit more participants. Advertisements were made in local press and candidates applied for the study through mail or phone. This effort resulted in 393 candidates who were assessed by phone; 181 were excluded by phone or after examination by physician. The remaining 212 were randomized. Another 18 patients were referred from another study in the clinic, and 12 of these met the inclusion criteria and were randomized after examination. Hence, 299 patients were randomized all together (Fig. 1): 99 in the ACT-bsm group, 100 in the Exercise group, and 100 in the CON group. Block randomization in groups of 30 patients was conducted to ensure equal size of the treatment groups despite consecutive recruitment. Hence, when a pool of 30 patients were recruited each of them were randomized at the same time to one of the three conditions. A statistician prepared an excel sheet where ten cells for each group were put in random order in the first column. The included participants were then put in the next column consecutively. University staff not otherwise involved in this study handled the randomization.

According to Swedish regulations and laws we had to present a sample size calculation in the ethical application. As reported at ClinicalTrials.gov this study had several primary outcomes. Our sample size calculation used several of these and we concluded in the ethical application that 70 persons in each intervention arm was needed. We also anticipated dropouts after randomization, which required that we recruited more subjects. We decided that 100 in each intervention arm was necessary to recruit. In Fig. 1 is shown that 73–81 subjects received the allocated intervention.

The study was approved by the regional ethical vetting board in Linköping (dnr: 2011/350–31). The study was also registered in Clinical Trials (Trial registration: ClinicalTrials.gov Id: NCT02399644).

### Procedure

All participants answered a questionnaire before the intervention (baseline) (T0), immediately after the intervention (T1), at a six-month follow-up (T2), and at a 12-month follow-up (T3).

### Outcomes

In this study, the following variables were used.

#### Insomnia severity index (ISI)

The primary outcome measure is the Insomnia Severity Index (ISI) and it was used for quantifying perceived insomnia severity [22, 23]. The seven items of ISI relate to the diagnostic criterion insomnia disorder and are rated on a 5-point scale (0–4). The scores of the seven items are summed to give a total ISI score (max = 28). The score is divided into four categories: no clinically significant insomnia (ISI: 0–7); sub-threshold insomnia (ISI: 8–14); moderate clinical insomnia (ISI: 15–21); and severe clinical insomnia (ISI: 22–28) [23]. For psychometric properties of the Swedish version of ISI in chronic pain patients, please see Dragioti, et al. [24]. No criteria were set for insomnia level with the aim of covering the entire cohort and of maintaining high ecological validity. This approach enables future sub group analyses due to the large sample size, but the reverse would have been impossible.

#### Pain intensity recent seven days

The average pain intensity for the most recent seven days (pain-7d) was registered on a 11-graded numeric rating scale (NRS) with endpoints of 0 (denoted no pain) and 10 (denoted the worst imaginable pain).

#### Hospital anxiety and depression scale (HADS)

The HADS, a short self-assessment questionnaire, measures symptoms of anxiety and depression. HADS comprises seven items in each of the depression (HADS-D) and anxiety scales (HADS-A) [25]. Possible subscale scores range from 0 to 21, the lower score indicating the least depression and anxiety possible. A score of 7 or less indicates a non-case, a score of 8–10 indicates a doubtful case, and a score of 11 or more indicates a definite case requiring further diagnostics [25]. HADS is frequently used for screening both in clinical practice and in research and has good psychometric characteristics [25, 26].

### Interventions

A group of any of the three conditions started when 7–10 patients were available.

For a certain group the time points (day/s of the week and time of the day) for the sessions were predetermined. But the next group had other time points (day/s of the week and time of the day) for their sessions; an assigned patient who could not participate in one group could wait some weeks and then participate in a group with more suitable time points for the sessions.

#### The physical exercise intervention (Exercise)

For eight weeks, the participants performed physical exercise in a group for one hour twice a week with two days in-between and at the same time points of the day. The sessions were held either at the clinic’s gym or at a nearby training facility. The exercise was supervised by a physiotherapist and each group had five to ten participants. A physician attended the first training session and informed the participants about the research on physical exercise and chronic pain. The training program included graded exercises. This means that the intensity and the load on the muscles increased gradually to increase the participants’ physical capacity over time. The exercises were primarily directed towards the neck and lower back regions. The participants also performed general strength training of the larger muscle groups, coordination exercises, and endurance exercises.

The training was split in two parts during the exercise hour. The first 30 min were performed as group exercise containing endurance, coordination, balance, functional strength, and movement training. After four weeks, the program increased aerobic training and decreased exercises that were directed towards range of movement. The goal was to achieve at least moderate intensity (i.e. just able to talk to other participants). The second part of the exercise session contained ten training stations and lasted for 30 min. This training included strength exercises for back, neck, abdomen, shoulders, and arms. For each exercise was defined three intensity levels and the intention was that the participant should increase the load throughout the intervention period and if possible achieve the highest level.

The participants were encouraged to do exercises at home one to two times a week for 30 min in the form of light-moderate aerobic exercise such as walking, swimming, or other aerobic exercises the participants found engaging. There was also an opportunity for the participants to do specific exercises due to problems in the neck or back. Every participant received an exercise diary where they recorded for each week which exercises they performed, how frequently they performed the exercises, and how long they performed the exercises. Once a week, the at home exercise sessions were followed-up in the group by the physiotherapist.

#### Acceptance and commitment therapy based stress management (ACT-bsm)

The course is originally a stress management program called Acceptance and Commitment Therapy at work (ACT at work) developed by Flaxman and co-workers [21]. The Swedish version, developed by Livheim, has been adapted to fit in a chronic pain setting and is here called Stress Management Intervention (ACT-bsm). The group treatment consists of seven weekly two-hour sessions and offers a mix of lectures and experience-based exercises such as role-play and mindfulness. A psychologist with special training in ACT-bsm led the group sessions of each course since the aim was to test the effect of the manual, and future studies will examine whether equal outcome can be achieved if other professionals (i.e. different professions) teach the course. In total three psychologists were involved in the ACT-bsm intervention throughout the study period to decrease the risk of a therapist effect. The sessions were held in a conference room at the hospital.

Because chronic pain is a major stressor [1], the initial focus was on the link between stress and chronic pain. A physician attended the first meeting to explain the biological processes involved in both conditions and why stress management makes sense for this group of patients. The second session focused on human language and why it is a source of suffering. Human language makes it possible to solve complex problems in our minds, but we can get stuck when we try to problem solve inner experiences such as thoughts and feelings. Language explains our ability to address threats that are not literally present, such as memories and catastrophizing about the future. Acceptance was emphasised as an alternative approach and mindfulness exercises were introduced as homework assignments.

Sessions three and four were devoted to valued life directions. These sessions were used to chart what is important in life in order to find guidance for future behaviour. If the impact of pain on behaviour is to decrease, it is helpful to be aware of what one would like to do instead. At the end of the fourth session, patients were given the opportunity to try yin-yoga as a complement to the mindfulness exercises. Yoga was used as a form of exposure therapy and the patients were instructed to stay in yoga positions for a couple of minutes while noticing what kind of emotions and thoughts they have. If they managed to make room for these experiences, they will be better suited to deal with other demanding situations in life despite the presence of discomfort.

Weeks five and six focused on behavioural change and the participants practiced strategies to deal with obstacles. The psychologist encouraged the patients to take steps in valued life directions and emphasised that memories and feelings related to earlier experiences are likely to show up. The last session emphasised communication and relationships to enhance assertiveness and to improve the quality of close relations. Lastly, the previous six sessions were briefly reviewed and some advice about how the participants could continue this work on their own was provided.

#### Control group (CON)

The active control group met once a week, two hours each time for seven weeks in a conference room at the hospital. On each occasion, there was one or more themes related to persistent pain to discuss the participants’ experiences. The group chose what to discuss at each session. Some examples of themes were how pain affects work, leisure, and relationships and their experiences with social insurance, employment services, and health care. A moderator (medical student, psychologist student, research nurse, or licenced psychologist) attended every session to help facilitate the discussions and to ensure that all group members were included in the discussions. The moderator consciously brought no active treatment intervention and encouraged the participants to decide the content of each session. If necessary, the moderator helped start the conversation by asking, for example, one of the following questions: "Does anyone have a similar experience?"; "Do you recognize this?"; "What do you usually do?"; and "What you said about that sounded interesting. Is there anyone who has had a similar experience?". The moderator acknowledged that their questions could make them feel vulnerable: "It's not easy"; "This can be hard to talk about this"; and "It's ok to go out and take a break if we feel we need to". To help mitigate conflicts in the group, the moderator made observations and asked the group questions: "It seems to be an important issue on which we have different experiences" and " Should we try to move on, maybe choose a new theme?". The moderator could also enhance the participants’ involvement in the group with comments such as "Interesting to hear" and "Thank you for sharing".

### Statistics

Basic statistical analyses were made using the statistical package IBM SPSS Statistics (version 23.0.0.2; IBM Corporation, Route 100 Somers, New York, USA). Data are presented as mean ± one standard deviation (± 1SD). For within group comparisons, paired samples t-test were used, comparing every measure point with baseline values. ANOVA and Tukey’s post-hoc test was used for between group comparisons. For bivariate correlation analysis was used the Pearson product-moment correlation coefficient.

For the mixed model analyses were used Stata version 15.0 (StataCorp LLC, College Station, Texas, USA). Test of data normality was performed using the Shapiro-Wilk test. Because the occasions (T0 to T3) are nested within individuals, we employed a 2-level mixed linear model with occasions (“time” at level 1) that are nested within individuals (level 2). The random part of the variance components model is individuals. The normality of the study outcomes was assessed using Shapiro Wilk test. The statistical analysis of each study outcome was performed using a mixed linear model with treatment group as fixed variable, with occasions nested within individuals, and an interaction of time by treatment group. The interaction between treatment group and time was tested using the likelihood ratio test. In the presence of an interaction, then the analysis was stratified by treatment group and a mixed linear model with occasions nested within individuals was performed separately for each group. In the absence of interaction, then we analysed the study outcome with a mixed linear model with treatment group as fixed variable and with occasions nested within individuals.

For each outcome variable was made two mixed models. The first analysis included only completers and the second analysis both completers and non-completers. Patients randomized but not at all participating in the interventions were excluded. Hence, the second analysis is a modified intention-to-treat analysis. Such a modified type of intention-to-treat analysis is frequently applied in the literature [27–29]. The reason for the exclusion of those randomized but not participating in the interventions was that this study focused upon the effects of interventions received and if they differed between the three conditions. Mixed model analysis handles missing data; no imputation method was applied since there are reports that imputations before a mixed model analysis is not necessary and simulations indicated that imputations before mixed models could result in unstable results [30].

Between-group effect sizes [31] were calculated based on the difference in mean change scores between each intervention and the control condition. This approach considers an eventual change in the control condition. Those values were divided by the baseline pooled standard deviation [32] from the entire sample to estimate the true standard deviation of the population as thoroughly as possible. This approach is referred to as d_ppc2_. Calculations were conducted using the webpage https://www.psychometrica.de/effect_size.html [33]. Cohen [34] describes an ES of .2 as small, .5 as medium, and .8 as large.

In all statistical tests, *p* < 0.05 (two-tailed) was considered significant.

## Results

### Dropouts immediately after randomization

There were dropouts after randomization (labelled *study non-completers*) in the three arms (ACT-bsm: 18%, Exercise: 22% and CON: 27%. Hence, the study non-completers never participated in the assigned interventions. Even though the percentage of study non-completers was higher in CON no group difference existed (Chi^2^ = 2.24; *p* = 0.33).

### Completers vs. treatment non-completers

Participants who were present at nine of 16 sessions for physical exercise and four of seven sessions for the other two group sessions were considered completers. The rationale for these cut-offs was based on what reasonable could be associated with physiological and/or psychological effects. This definition of completer resulted in 65 completers in the Exercise group, 60 completers in ACT-bsm group, and 60 completers in the CON group (Fig. 1). Those who did not fulfil the criteria of completers are labelled *treatment non-completers*. A Chi^2^ test revealed that the distribution between completers/study non-completers/treatment non-completers did not differ between the treatment arms (Chi^2^ = 4.82, *p* = 0.31).

No significant differences in proportion of treatment non-completers vs. completers were found (Chi^2^ = 2.50; *p* = 0.29) between the three intervention arms (ACT-bsm: 26%, Exercise: 17% and CON:18%).

No pre-treatment (T0) differences were found between completers and treatment non-completers for age, ISI, Pain-7d, HADS-A or HADS-D (Table 1).

**Table 1:** Age and outcomes (mean +/- one standard deviation (SD)) in Completers vs. treatment non-Completers at baseline (T0). Furthest to the right is the statistical comparison (ANOVA; *p*-value)

Variable	Completers (*n* = 183–185)	Treatment non-Completers (*n* = 40–42)	Statistics
	Mean ± SD	Mean ± SD	p-value
Age	54.21 ± 10.15	54.08 ± 11.03	.940
ISI	13.42 ± 6.65	12.19 ± 7.44	.290
Pain-7d	5.81 ± 1.95	5.93 ± 1.90	.737
HADS-A	7.90 ± 4.23	7.41 ± 5.08	.510
HADS-D	6.17 ± 3.69	5.29 ± 4.12	.170

*ISI* Insomnia Severity Index, *Pain-7d* pain intensity recent 7 days according to 11-graded numeric rating scale, *HADS-A* Hospital Anxiety and Depression Scale subscale Anxiety, *HADS-D* Hospital Anxiety and Depression Scale subscale depression

### Completers at baseline – comparisons between the three intervention arms

There were no significant differences between the three groups at T0 neither for age (ACT-bsm: 53.8 ± 10.6 years, Exercise: 54.5 ± 10.0 years, CON 54.3 ± 10.0 years; *p* = 0.933) nor for ISI (*p* = 0.479), Pain-7d (*p* = 0.877), HADS-A (*p* = 0.318) and HADS-D (*p* = 0.138) (for figures see Table 2a).

**Table 2:** Mixed model analyses of completers (a) and for completers and treatment non-completers taken together (intention to treat; b) for the four outcomes: ISI, Pain-7d, HADS-anxiety and HADS-depression

		Exercise	Within grp p-value	ACT-bsm	Within grp p-value	CON	Within grp p-value	Time vs T_0_ p-value
a) Completers								
ISI								
Time x group (p-value)	.037							
T_0_		13.48 ± 6.52	ref	14.23 ± 6.00	ref	12.76 ± 7.24	ref	–
T_1_		12.14 ± 6.55	.020	13.25 ± 6.30	.071	13.15 ± 7.46	.234	–
T_2_		11.64 ± 7.15	.002	13.24 ± 6.38	.215	11.53 ± 7.29	.122	–
T_3_		11.19 ± 6.27	.001	12.22 ± 6.38	.009	12.59 ± 7.13	.851	–
Pain-7d								
Time x group (p-value)	.003							
T_0_		5.91 ± 1.77	ref	5.78 ± 1.97	ref	5.73 ± 2.15	ref	–
T_1_		4.58 ± 2.14	.001	5.45 ± 1.99	.142	5.67 ± 2.15	.930	–
T_2_		5.14 ± 2.36	.015	5.59 ± 2.24	.315	5.02 ± 2.02	.010	–
T_3_		5.09 ± 2.17	.011	5.47 ± 1.91	.169	5.04 ± 2.22	.025	–
HADS-A								
Overall effect of time (p-value)		.003		.017		.276		
Overall effect of group (*p*-value)		.211		.916		ref		
Time x group (p-value)	.112							
T_0_		8.56 ± 4.42	ref	7.52 ± 4.14	ref	7.60 ± 4.10	ref	ref
T_1_		7.08 ± 4.79	–	6.77 ± 3.77	–	7.42 ± 4.54	–	<.001
T_2_		7.17 ± 4.26	–	6.59 ± 3.76	–	5.85 ± 4.45	–	<.001
T_3_		7.30 ± 4.46	–	6.56 ± 3.90	–	7.27 ± 3.80	–	.001
HADS-D								
Overall effect of time (p-value)		.137		.011		.487		
Overall effect of group (p-value)		.719		.260		ref		
Time x group (p-value)	.197							
T_0_		6.40 ± 3.65	ref	6.78 ± 3.37	ref	5.48 ± 3.94	ref	ref
T_1_		5.41 ± 3.86	–	6.07 ± 3.80	–	5.42 ± 3.82	–	.002
T_2_		5.52 ± 3.27	–	6.04 ± 3.61	–	4.87 ± 3.75	–	.005
T_3_		5.67 ± 3.90	–	5.56 ± 3.75	–	5.49 ± 3.75	–	.037
b) Completers + treatment non-completers								
ISI								
Time x group (p-value)	.015							
T_0_		13.48 ± 6.49	ref	13.73 ± 6.16	ref	13.14 ± 7.31	ref	–
T_1_		11.63 ± 6.50	.001	13.39 ± 6.47	.260	13.39 ± 7.45	.216	–
T_2_		11.83 ± 7.29	.003	12.98 ± 6.16	.594	11.73 ± 7.27	.055	–
T_3_		11.17 ± 6.22	.002	12.32 ± 6.19	.057	12.49 ± 7.01	.955	–
Pain-7d								
Time x group (p-value)	.009							
T_0_		5.88 ± 1.78	ref	5.84 ± 1.96	ref	5.86 ± 2.11	ref	–
T_1_		4.64 ± 2.29	.000	5.59 ± 1.99	.105	5.52 ± 2.13	.355	–
T_2_		5.14 ± 2.28	.020	5.57 ± 2.31	.228	4.92 ± 2.05	.002	–
T_3_		4.98 ± 2.30	.004	5.39 ± 2.03	.057	4.94 ± 2.25	.003	–
HADS-A								
Overall effect of time (p-value)		.024		.021		.089		
Overall effect of group (p-value)		.784		.337		ref		
Time x group (p-value)	.103							
T_0_		8.22 ± 4.58	ref	7.41 ± 4.09	ref	8.01 ± 4.66	ref	ref
T_1_		6.84 ± 4.79	.000	7.02 ± 3.91	.218	7.85 ± 4.82	.582	.001
T_2_		7.09 ± 4.40	.002	6.64 ± 3.71	.033	6.15 ± 3.88	.000	<.001
T_3_		7.21 ± 4.73	.021	6.63 ± 4.13	.036	7.17 ± 3.78	.475	.003
HADS-D								
Overall effect of time (p-value)		.303		.065		.931		
Overall effect of group (p-value)		.355		.434		ref		
Time x group (p-value)	.494							
T_0_		6.32 ± 3.75	ref	6.20 ± 3.45	ref	5.75 ± 4.21	ref	ref
T_1_		5.37 ± 3.90	.006	5.94 ± 3.75	.025	5.57 ± 3.69	.721	.002
T_2_		5.58 ± 3.44	.058	5.81 ± 3.61	.198	5.08 ± 3.72	.396	.018
T_3_		5.73 ± 3.99	.297	5.53 ± 3.75	.042	5.42 ± 3.73	.739	.096

*Exercise* exercise intervention, *ACT-bsm* Stress Management Intervention, *CON* controls, *ISI* Insomnia Severity Index, *Pain-7d* pain intensity recent 7 days according to 11-graded numeric rating scale, *HADS-A* Hospital Anxiety and Depression Scale subscale Anxiety, *HADS-D* Hospital Anxiety and Depression Scale subscale depression, *T0* baseline, *T1* immediately after the intervention, *T2* 6-month follow-up, *T3* 12-month follow-up

### Mixed model analyses

#### ISI

##### Completers

There was no statistically significant departure from normality as tested by Shapiro-Wilk test. Because the interaction of time by treatment group was statistically significant (*p* = 0.037) (Table 2a), the analysis was stratified. The mixed analysis of ISI with time points nested within subjects was performed separately for each treatment group.

**Fig. 2:**
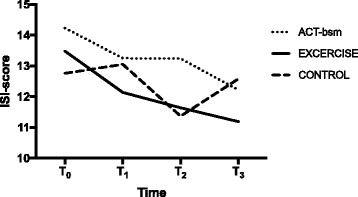
ISI (mean) for each treatment arm (completers) before treatment (T0), after treatment (T1), after six months (T2), and after twelve months (T3)

The fixed-effect estimates showed no statistically significant effect of time in the control group (*p* = 0.622). The fixed effect estimates yield a negative slope that was statistically significant both in ACT-bsm (*p* = 0.017), and in Exercise (*p* = 0.001) (Table 2a). In ACT-bsm, the decrease over time was significant in T3 vs. T0 (*p* = 0.009), but not for the other time points (T1 vs. T0: *p* = 0.071; T2 vs. T0: *p* = 0.215). In Exercise, ISI decreased significantly at all time points compared with T0 (T1 vs. T0: *p* = 0.020; T2 vs. T0: *p* = 0.002; T3 vs. T0: *p* = 0.001). In Fig. 2 is shown ISI in the three intervention arms at the four timepoints.

##### Completers + treatment non-completers

The interaction of time by treatment group was statistically significant (*p* = 0.015) (Table 2b), therefore we stratified the analysis. The mixed analysis of ISI with time points nested within subjects was performed separately for each treatment group.

There was no statistically significant decrease of ISI neither in CON nor in the ACT-bsm. In Exercise, the decrease over time was statistically significant at all time points compared with T0 (T1 vs. T0: p = 0.001; T2 vs. T0: *p* = 0.003; T3 vs. T0: p = 0.002) (Table 2b).

#### Pain intensity- pain-7d

##### Completers

**Fig. 3:**
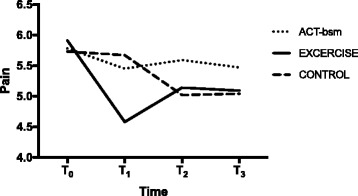
Pain-7d (mean) for each treatment arm (completers) before treatment (T0), after treatment (T1), after six months (T2), and after twelve months (T3)

There was no statistically significant departure from normality. Because the interaction of time by treatment group was statistically significant (*p* = 0.003) (Table 2a), the analysis was stratified. The mixed analysis of Pain-7d with time points nested within subjects was performed separately for each treatment group. The decrease of Pain-7d was statistically significant in CON (T2 vs. T0: *p* = 0.010; T3 vs. T0 *p* = 0.025), in Exercise (T1 vs. T0: *p* < 0.001; T2 vs. T0: *p* = 0.015; T3 vs. T0: *p* = 0.011), but not in ACT-bsm (Table 2a, Fig. 3).

##### Completers + treatment non-completers

The interaction of time by treatment group was statistically significant (*p* = 0.009) (Table 2b), therefore we stratified the analysis. The mixed analysis of Pain-7d with time points nested within subjects was performed separately for each treatment group.

There was no statistically significant decrease of Pain-7d in the ACT-bsm. In CON, the decrease over time was not statistically significant at T1 vs. T0 (T1: *p* = 0.355), but was significant at T2 (T2: *p* = 0.002) and T3 (T3 vs. T0: p = 0.003) (Table 2b). In Exercise the decrease was significant at all time points vs. T0 (T1 vs. T0. *p* < 0.001; T2 vs. T0: p = 0.02; T3 vs. T0: *p* = 0.004).

#### HADS-A

##### Completers

There was some statistically significant departure from normality as tested by Shapiro-Wilk test (Control T1 and Exercise group T1 and T2: p = 0.02). The interaction of time by treatment group was not statistically significant (*p* = 0.112). In the analysis with the main effects of treatment group and time, there was no statistically significant difference between groups (ACT-bsm vs. control: *p* = 0.575; Exercise vs. control: *p* = 0.768) (Table 2a). The decrease of HAD-anxiety over time was highly statistically significant at all time points (T1 vs. T0 and T2 vs. T0: *p* < 0.001; T3 vs. T0: *p* = 0.001) (Table 2a).

When data was transformed (log (X + 1)) the departure from normality increased.

When we compared the changes in HADS-A from T0 to T3 between the three groups no significant differences existed (ACT-bsm: − 0.89 ± 3.22, Exercise: − 1.42 ± 3.45, CON: -0.16 ± 2.99; *p* = 0.135); similar results were obtained when the changes from T0 to T1 and from T0 to T2 were analysed (data not shown).

##### Completers + treatment non-completers

The interaction of time by treatment group was not statistically significant (*p* = 0.10) (Table 2b). In the analysis of main effects of treatment group and time, there was no difference between groups (ACT-bsm vs. CON: *p* = 0.337; Exercise vs. CON: *p* = 0.784) (Table 2b). The decrease over time compared with baseline (T0) was statistically significant at all time points vs. T0: T1 (T1 vs. T0: p = 0.001; T2 vs. T0: p < 0.001; T3 vs. T0: *p* = 0.003).

#### HADS-D

##### Completers

There was statistically significant departure from normality (as tested by Shapiro-Wilk test): in CON at all time points (T0: *p* = 0.002; T1: *p* = 0.01; T2: p < 0.001; T3: *p* = 0.02), in ACT-bsm at T3 (p = 0.01), and in the exercise group at T2 (p = 0.02).

The interaction of time by treatment group was not statistically significant (*p* = 0.197) (Table 2a). In the analysis with the main effects of treatment group and time, there was no statistically significant difference between groups (ACT-bsm vs. CON: *p* = 0.260; Exercise vs. CON: *p* = 0.719). The decrease of HAD-Depression over time was significant at all time points (T1 vs. T0: *p* = 0.002; T2 vs. T0: *p* = 0.005; and T3 vs. T0: *p* = 0.037) (Table 2a).

When data was transformed (log (X + 1)) it was still departing from normality. Similar results were obtained as in the models using non-transformed data.

When we compared the changes in HADS-D from T0 to T3 between the three groups no significant differences existed (ACT-bsm: − 1.02 ± 3.12, Exercise: − 0.93 ± 3.94, CON: + 0.25 ± 2.27; *p* = 0.088); similar results were obtained when the changes from T0 to T1 and from T0 to T2 were analysed (data not shown).

##### Completers + treatment non-completers

The interaction of time by treatment group was not statistically significant (*p* = 0.49) (Table 2b). In the analysis of main effects of treatment group and time, there was no difference between groups (ACT-bsm vs. CON: *p* = 0.786; Exercise vs. CON: *p* = 0.781) (Table 2b). The decrease over time compared with baseline (T0) was statistically significant at T1 (T1 vs. T0: p = 0.002) and T2 (T2 vs. T0: *P* = 0.018), but was not significant at T3 (T3 vs. T0: *p* = 0.096).

##### Effect sizes for ISI and pain-7d

The effect sizes for the ISI and pain-7d are shown in Table 3. The effect sizes (i.e., d_ppc2_) indicated small to medium size effects for the ISI for Exercise at T1 and T3 compared to baseline. Compared to baseline, the d_ppc2_ for pain-7d in the Exercise group were medium to large compared to CON at T1.

**Table 3:** Effect sizes for significant group x time interactions (p < 0.05) for completers only and for completers together with treatment non-Completers (Intention to treat) based on pooled standard deviation. The two conditions were compared with the active controls (CON)

	Exercise		ACT-bsm	
	Completers	Intention to treat	Completers	Intention to treat
ISI (d_ppc2_)*				
T_1_	−.262	−.318	−.207	−.089
T_2_	−.092	−.038	.036	.100
T_3_	−.321	−.252	−.279	−.115
Pain-7d (d_ppc2_)*				
T_1_	−.650	−.463	−.138	.046
T_2_	−.031	.103	.266	.345
T_3_	−.067	.010	.194	.242

*ISI* Insomnia Severity Index, *Pain-7d* pain intensity recent 7 days according to 11-graded numeric rating scale, *T0* baseline, *T1* immediately after the intervention, *T2* 6-month follow-up, *T3* 12-month follow-up. * [33]

##### Correlations

Weak correlations were found between change in pain-7d and change in ISI (T0 vs. T1) in Exercise (Pearson *r* = .445, *p* < .01) and in all three groups taken together (Pearson *r* = .269, p < .01).

## Discussion

This study evaluated the effects of physical exercise (Exercise) and stress management (ACT-bsm) on insomnia in patients suffering chronic pain. The mixed model analyses revealed that Exercise had a positive effect on insomnia compared with CON and the effect remained after 12 months. No clear effect (i.e., both for completers and for completers together with treatment non-completers) upon ISI was found for ACT-bsm.

Changes on the ISI were small but significant for Exercise compared with CON (Table 2a and b). The clinically important change in ISI is not well established. By comparing ISI scores with global improvement ratings from an independent assessor (corresponding moderate change), Morin, et al. [23] found a minimally important difference of > 7 points in treatment studies on primary insomnia. The mean change scores in the exercise group in this study is 2.32 after 12 months, which is below the clinical importance threshold according to Morin et al. Nevertheless, our results are about the same magnitude as those reported by Wilson, et al. [4]. Notably, they had no follow-up and slightly higher baseline values (16.08 vs. 13.48 in the present exercise group). Moreover, their intervention was multimodal including both CBT and a lecture about sleep hygiene. There were also differences in duration and dose as Wilson and co-workers trained their subjects four times a week for three weeks (compared to two times a week for eight weeks in this study). In another relevant study, Daly-Eichenhardt, et al. [35] define clinically significant change of ISI in a pain context as a change of at least half a standard deviation. In our study, that would mean a change score of 3.33 according to ISI based on the pooled standard deviation from all three treatment groups at baseline, which the threshold was also one point above the ISI improvement in our study. Notably, Daly-Eichenhardt, et al. [35] included two sleep-specific sessions in their group-based MMRP and two extra individual sessions for patients in need of sleep compression. The mean baseline values for ISI in that study exceeded 20 points and in that perspective an improvement of 2.91 points (half their standard deviation) is also rather modest. Mean change after nine months was 2.22 points compared to baseline. Moreover, the study lacked a control condition. In summary, our data concerning changes of ISI agree with other studies. There is a need to establish clinically important changes for ISI. The clinical importance of the moderate beneficial effect of Exercise on pain-related insomnia measured by ISI may need further investigation.

The reduction in pain intensity (pain-7d) was significant both for Exercise and CON but not for ACT-bsm. Reductions in pain intensity have been reported for different types of exercise interventions in patients with chronic pain. The reason for the un-changed pain intensity in ACT-bsm is not apparent. Changes in ISI correlated weakly with changes in pain intensity levels in Exercise (T0 vs. T1) but not in the two other intervention arms. The decrease in pain-7d could be part of the explanation for improved sleep in Exercise. The effect on pain-7d in the exercise intervention was somewhat reduced over time and no correlations existed between changes in ISI and pain-7d at T2 and T3; the positive change in sleep continued to increase over the 12 months in Exercise. Hence, other factors than pain intensity may reasonably mediate the positive effects of exercise on ISI as well. Another explanation for the superior positive effects for ISI in Exercise vs. other arms could be that exercise increased physiological fatigue and thereby had positive effects on sleep. Unfortunately, the study design did not include outcome variables that could have been used to analyse such a suggestion. However, even though significant and positive effects was found upon Pain-7d in Exercise and CON the changes were small and did not reach a clinically important change i.e., a reduction of at least 2 units or 30% according to NRS.

The literature is very limited with respect to the relationship between physical exercise and improvements in sleeping problems (i.e., insomnia) and therefore there are no established levels of optimal frequency, duration, and intensity of physical exercise to enhance sleep in patients with chronic pain conditions. Sixteen bi-weekly sessions could possibly be too little in the perspective of the relatively small improvements in ISI and pain-7d. Reid, et al. [10], who exercised healthy older adults for 64 sessions over 16 weeks, found large effect sizes but the sample size was small (*N* = 17). Thus, it is unclear whether that treatment would have the same effect in a larger sample of middle-age patients with chronic pain and if our study would show greater effect on insomnia with increased number of exercise sessions.

For the psychological variables (HADS-A and HADS-D) were found significant improvements over time but no group differences. The three intervention groups were below the cut-off levels indicating definite cases at T0 and it may then be difficult to achieve group differences. The reductions in HADS-A and HADS-D could have been due to regression to the mean. However, for both exercise and ACT influenced interventions have been reported significant effects – even though the literature not is in consensus - upon these variables in patients with chronic pain conditions.

### Strengths and limitations

Most studies in this field lack either randomization or control condition or both. This study – a RTC with a large sample size – contributes to the growing body of knowledge. Follow-ups at six and twelve months strengthens the results even further. The study sample and interventions contribute to high ecological validity in terms of comorbidity, gender, and dose. A limitation, which might reduce the external validity, is related to how patients were recruited. Even though there were different origins the major part (> 70%) was recruited using advertisements in the local press.

In all three interventions were found drop-outs after randomization (i.e. study non-completers) before start of the intervention. It is a limitation that reasons for these dropouts not were registered but dropouts could have been due to practical issues, lack of motivation and hopes of being randomized to a certain intervention. The somewhat higher proportion of study non-completers in CON than in the two active arms might have been due to lower credibility and expectations of effectiveness for this intervention. The research field of behavioural treatments struggle with the issue of un-blinded studies. This could partially be handled by including a treatment credibility measure in the pre and post measurements to assess if the control condition is preserved as credible as the other conditions [36]. The only indication available in this study for this limitation is the equivalent proportions of non-completers in all three groups. Although “intent-to-treat” analyses typically include all who are randomized our approach excluded those how never started an intervention. This was done since this study focused upon the effects of interventions received and if they differed between the three conditions. Such a modified type of intention-to-treat analysis is frequently applied in the literature [27–29].

In this study we have used cut-off values for participation to define completers/treatment non-completers. A limitation is that these were based on clinical experiences on what exposures are necessary to obtain changes. However, we achieved very similar results whether the treatment non-completers were included in the mixed model analyses (i.e., intention to treat analysis) or not. The reasons for being a study non-completer could be similar to those mentioned above for study non-completers (drop-outs) but could also include that the participant grasped the core content quickly and was able to use the intervention without further support and participation in the intervention groups.

Only one variable capturing sleeping problems (i.e., ISI) was included. Hence, the results concerning sleeping problems relies on one subjective measure. Sleep diaries or objective measures such as actigraphy could contribute with valuable data about sleep latency, wake time after sleep onset, and sleep efficiency. Actigraphy could also measure daytime activity in a reliable way. Moreover, we do not know about the distribution of sleep medications in the treatment arms.

Thus, future studies should examine several aspects of sleeping problems, and data on concomitant medication need to be captured.

There has been a growing interest in studying aversive events in non-pharmacological research in recent years [37]. It can be assumed that ACT-bsm will lead to a short-term increase of anxiety/stress because of an increased focus on negative private events. Furthermore, exercise can lead to increased pain levels due to movements of painful areas of the body and possibly delayed onset muscle soreness. This kind of information would be valuable for evaluating the utility of these treatments.

### Possible clinical implications

The treatment effects for ISI in the exercise intervention present study were statistically significant, but did not reach clinical significance according to definitions presented in other relevant studies. The results could be interpreted as beneficial side-effects on sleep caused by physical exercise. It strengthens the view that non-specific treatments that do not target sleep per se are not enough to remedy sleeping problems in most patients with chronic pain conditions. Treatments/strategies addressing sleep disturbances should be an active part of the multimodal treatment portfolio of chronic pain management. The most promising results for treating sleeping problems comorbid with chronic pain points to cognitive behavioural therapy specific for insomnia [38, 39]. The rapid development of and increased access to electronic devices presents new ways to administer treatment and collect sleep data.

## Conclusion

This study evaluates the effects of physical exercise and ACT based stress management with respect to insomnia in chronic pain patients. Beneficial significant effects on insomnia was confirmed in the exercise condition. However, these changes were probably not clinically important. For pain intensity a general decrease was found in the Exercise condition and in the control condition, while no change occurred in the ACT- based stress management condition. No condition differences were found for the two psychological variables.

## Abbreviations

ACT: Acceptance and Commitment Therapy
ACT-bsm: ACT based stress management
ANOVA: ANalysis Of VAriance
CBT: Cognitive Behavioural Therapy
CBT-I: Cognitive Behavioural Therapy for Insomnia
Comp: Completer
CON: Control group
Dnr.: Diary number
d_ppc2_: Effect size according to Morris [31]
HADS: Hospital Anxiety and Depression Scale
ISI: Insomnia Severity Index
MMRP: Multimodal/Multidisciplinary Rehabilitation Programmes
NRS: Numeric Rating Scale
Pain-7d: Pain intensity for the most recent seven days
Post: After treatment
Pre: Before treatment
RCT: Randomized Controlled Trial
SD: Standard Deviation
T0: Time point 0 = baseline
T1: Time point 1 = immediately after the intervention
T2: Time point 2 = at 6 month follow-up
T3: Time point 3 = at 12 month follow-up
